# Shorter Night Splinting by a Modified Ponseti Method With Similar Foot-Function and Health-Related Quality of Life

**DOI:** 10.7759/cureus.80260

**Published:** 2025-03-08

**Authors:** Angela Seidel, Severine Tinembart, Nadine Kaiser, Kai Ziebarth

**Affiliations:** 1 Departement of Orthopedic Surgery and Traumatology, University of Fribourg, Fribourg, CHE; 2 Department of Pediatric Surgery, Inselspital, University of Berne, Berne, CHE

**Keywords:** club foot, disease specific instrument, long-term outcome, ponseti method, quality of life

## Abstract

Background

The Ponseti method is the most frequent method for clubfoot treatment. It includes a six-year night-splinting period, which is difficult to comply with. The objective of this study is to evaluate the clinical outcomes, recurrence rates, and patient-reported health-related quality of life following a modified Ponseti method with reduced night splinting duration.

Methods

We analyzed 107 children (77 boys, 30 girls, 152 clubfeet) who were treated for idiopathic clubfoot from January 1994 to January 2015. The initial treatment started at a mean age of 9.2 days. Long-leg Soft Casts TM3 were applied until the desired position of the foot was achieved. At a mean age of 3.2 months, the residual deformity was corrected surgically. We assessed the clinical outcome by chart review and the functional outcome by a questionnaire that included the disease specific instrument (DSI) and the health-related quality of life assessed by the pediatric quality of life inventory (PedsQL™).

Results

In 101 patients (92.7%) and 142 feet (93.4%), we had a clinical follow-up at a mean age of 7.39±4.9 years. A Denise Brown splint or a unilateral orthosis was applied for a mean period of 7.8±4.8 months over the whole day and an additional 17.3 ±16.3) nights only. At a mean follow-up of 10.0±SD 6.2 years, 82.2% of patients returned the questionnaires. The mean DSI was 74.8±17.5. The mean overall PedsQL™ was 87.8±12.6. The PedsQL™ functional component score showed a mean of 89.4±5.6, and the PedsQL™ social component score a mean of 86.9±13.5. Ten (9.3%) patients had a bilateral, and 18 (16.8%) patients had a unilateral relapse.

Conclusions

Achilles tendon lengthening (ATL) and limited posterior release, followed by a reduced period of splinting of 2.1 years, provide similar results compared to the original Ponseti method.

## Introduction

Ponseti treatment for clubfeet is the established method all over the world [1]. Starting in the first days of life, serial specific manipulations and long leg casting are used to correct the deformity, followed by a percutaneous Achilles tendon lengthening (ATL)[1]. Afterwards, a Denis Browne splint has to be worn the whole day for a period of five months and at night for five to six years following the ATL [1]. Since the Ponseti method was easy to learn and good results were reported [2], it became state of the art [3]. However, to minimize the risk of recurrence, alternative treatment strategies as well as modifications of the original Ponseti methods, have been described [4,5].

The risk of recurrence correlates with the severity of the original deformity and noncompliance in wearing the splint [6]. Our experience, supported by literature, is that many parents have difficulty convincing their children to wear the device for the entire recommended time period [7,8]. Aiming to decrease the splinting duration, we started to perform a minimal open ATL followed by a posterior release in severe cases.

In our study, we aimed to analyze the duration of night splinting, the clinical outcome, and functional and health-related quality of life when following this modified Ponseti treatment with reduced night splinting duration.

## Materials and methods

Data selection

**Figure 1:**
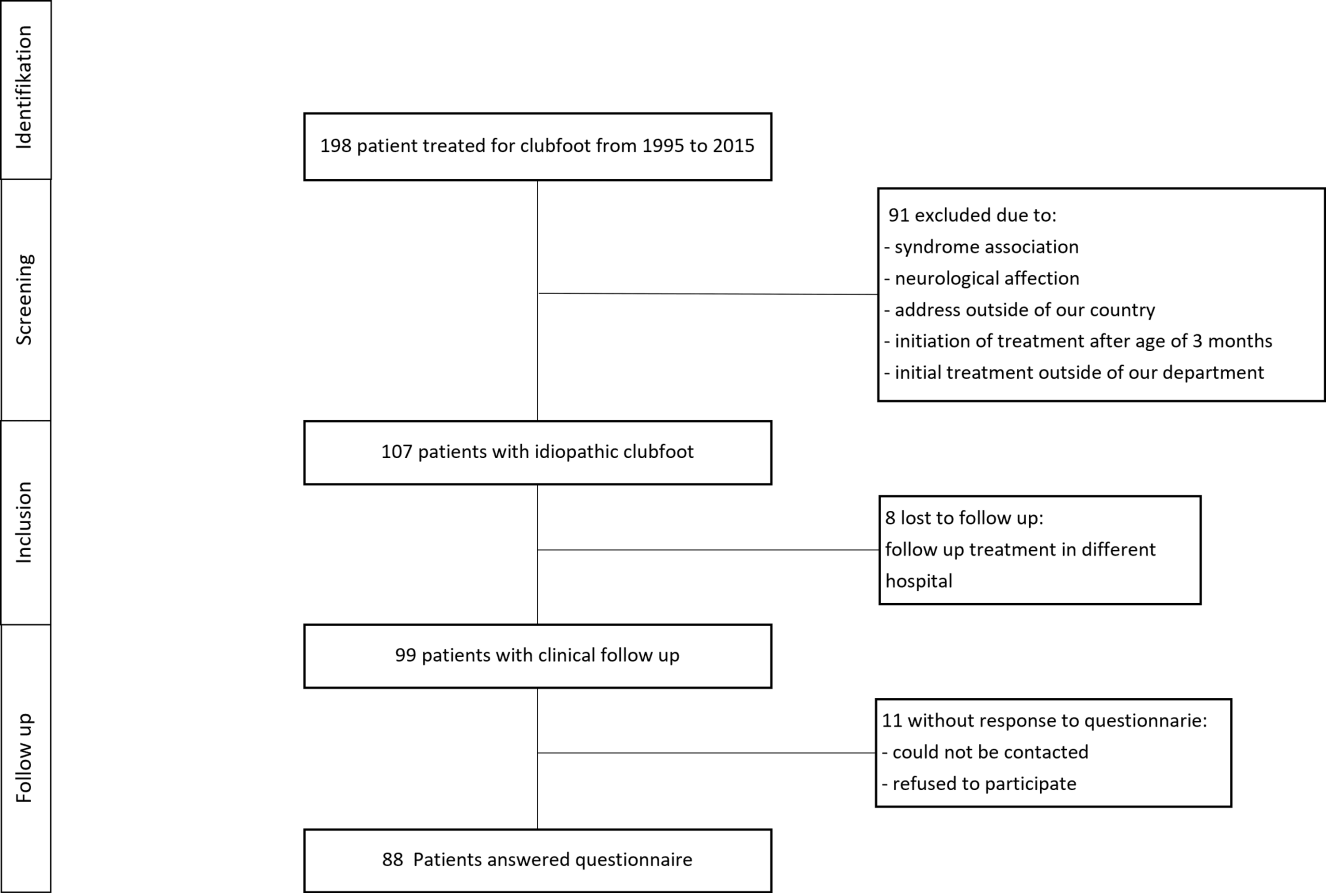
STROBE flow chart. Patients were included in this study according to the STROBE flow chart.

This is a retrospective chart review of patients treated for idiopathic clubfoot at our institution from January 1994 to January 2015. We identified all eligible patients using our hospital information system and excluded patients according to Figure 1. Written informed consent was obtained from all patients or guardians after the ethics review board approved the study (Kantonale Ethikkommission Bern (KEK) number 2016-01033).

From the patient charts, we recorded total splinting time, relapse rate, and range of motion (ROM). The splinting started six weeks after the operation and directly after removing the cast. The end of the splinting time was decided in the follow-up control. We individually classified the ROM of the ankle and subtalar joints. For the ankle, normal was defined as dorsiflexion greater than 15-degree; good as neutral dorsiflexion; and poor as dorsiflexion less than neutral. For the subtalar joint, we assumed a baseline 20-degree motion and defined normal as 66%-100% of baseline, good as 33%-66%, and poor as less than 33% [9].

From 2018 to 2022, we sent questionnaires to the patients or guardians. It included the disease specific instrument (DSI) [10] and a health-related quality of life assessed by pediatric quality of life inventory (PedsQL™) [11]. As the primary outcome measure, we have chosen the DSI, which is a ten-item validated questionnaire. Responses were recorded on a 4-point Likert scale (eg, very satisfied, somewhat satisfied, somewhat dissatisfied, very dissatisfied). The scores were standardized to a score of 0 to 100, with higher scores indicating better outcomes. For the analysis of self-reported health-related quality of life, we have selected the pediatric quality of life as a secondary outcome, of which a validated German version is available. The scores were standardized to a score of 0 to 100, with higher scores indicating a better quality of life.

Clubfoot classification

Until 2012, the treating surgeon graded each clubfoot. The system ranged from 1 (mild) to 6 (rigid). Starting in 2012, the Pirani score was introduced for all patients [12]. A mild score corresponds to a Pirani score of 1-1.5, mild to moderate to 2-2.5, moderate to 3-3.5, moderate to severe to 4-4.5, severe to 5-5.5, and a rigid score equates to a Pirani score of 6.

Treatment and surgical technique

**Figure 2:**
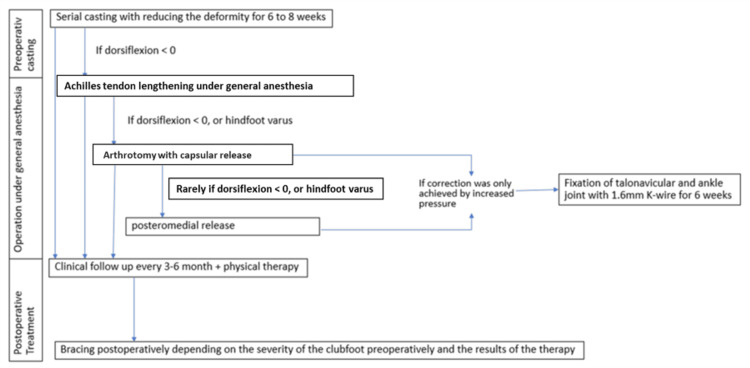
Treatment plan. Flow chart of the treatment plan including intraoperative decision making, made by authors.

According to the Ponseti protocol [1], we gradually reduced the clubfoot deformity over 6-8 weeks with serial casting. If the foot was in an acceptable position but still demonstrated equinus, Achilles tendon lengthening (ATL) was scheduled under general anesthesia (Figure 2).

**Figure 3:**
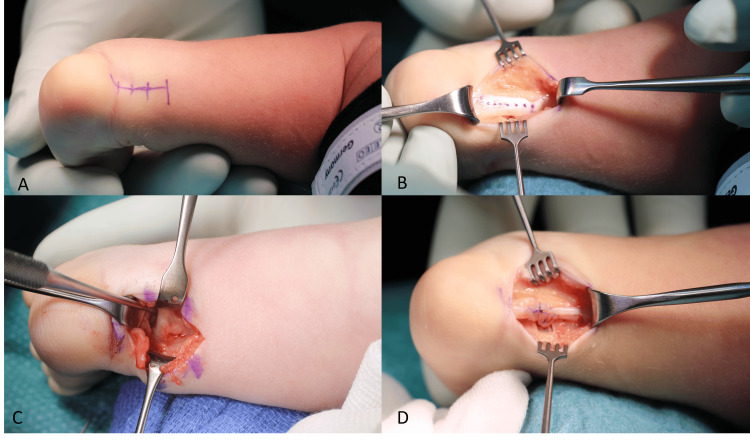
Intraoperative imaging. A. The incision is planned at the medial side of the Achilles tendon slightly proximal to its insertion; B. The Achilles’ tendon lengthening is performed in a z; C. If the dorsiflexion of the ankle was limited to neutral or the hindfoot was in varus, we added a limited posterior release of the ankle joint capsule, was performed in D Image after suture of the Achilles tendon.

The skin was incised at the medial border of the Achilles tendon to perform a z-lengthening (Figure 3).

The ROM of the ankle and subtalar joints were tested. If the dorsiflexion of the ankle was limited to neutral or the hindfoot was in varus, we added a limited posterior release (Figure 3). We used the interval between the flexor hallucis and the peroneal muscle to perform the arthrotomies of the ankle and subtalar joints. The talus was mobilized and centralized under the tibia. Rarely, in very severe and rigid cases, an advancement of the incision to the medial forefoot was added. If needed, a lengthening of the flexor tendons (flexor hallux longus, flexor digitorum longus, and tibialis posterior) was performed. The aim of the procedure was 20° dorsiflexion and 50° external rotation of the foot. The forefoot had to be aligned in a neutral position. If dorsiflexion or external rotation was only achieved by increased pressure, the talonavicular and ankle joint were temporarily fixed with 1.6mm K-wires. Immobilization was accomplished in an above-knee cast for six weeks postoperatively.

Depending on the clinical findings, we applied a Denis Browne brace or a unilateral above-knee orthosis for 3-6 months, day and night. In the first year, we examined the feet every three months. In uneventful cases, we advanced the visits to 6 months. A physiotherapist attended all patients during the first 1-2 years. We weaned out the braces after 2-3 years postoperatively, depending on the initial severity of the clubfoot and the results of the therapy.

Statistical analysis

For statistical calculations, SPSS statistics (IBM SPSS Statistics for Windows, Version 21.0) were used. Continuous data (DSI, Peds-QL score) were tested for normal distribution with the Kolmogorov-Smirnov test. Descriptive data was assessed by means and standard deviation. Risk factor analysis was performed with multiple linear regression. To calculate the effect size of the difference of our DSI score with the literature, we used Cohen's d.

## Results

Descriptive data

Using the above-mentioned criteria, 107 patients (77 boys, 30 girls, 152 clubfeet) were eligible to be included in the study. As those patients were regularly seen in the outpatient clinic, the data of those patients could be used for the descriptive data, postoperative splinting time, and relapse rate. In 45 children, both feet were affected, in 34 children only the right, and in 28 children only the left foot was treated.

These patients had the following distribution of the severity score: mild, 10; mild to moderate, 12; moderate, 32; moderate to severe, 25; severe, 39; and rigid, 34. In 23 patients (21.5%) and 33 feet (21.7%), a Pirani score was documented with a mean score of 4.10±1.0.

Ponseti casting started at a mean age of 9.2±7.9. At a mean age of 3.2±2.3 months, patients underwent open ATL and limited posterior release. We had no immediate postoperative complications that led to a secondary intervention.

Postoperative splinting time

Seventy-four patients (69.2%; 104 feet: 68.4%) received an orthosis, and 33 patients (30.8%; 48 feet: 31.6%) got a Denis Browne splint. These splints were worn for the whole day over a mean period of 7.8±4.8 months and for nights only over an additional 17.3 ±16.3 months. More detailed information can be found in Table 1.

**Table 1:** Postoperative splinting and outcome: questionnaire. Outcome, operative and postoperative treatment listed stratified by the initial severity of the clubfoot. The disease specific instrument (DSI) and the pediatric quality of life inventory (PedsQL™) are the mean scores of the group of the feet (n=x) available at follow-up.

Classification	Feet N (%)	Type of splint orthesis/ Denis Browne splint N (%)	Splint applied the whole day (mean in month±SD)	Splint applied nights only (mean in month±SD)	Follow-up for questionnaires (in years±SD)	DSI	PedsQL™
Mild	10 (6.6%)	6/4 (60/40%)	10.5±2.8	30.9±28.2	6.0±2.5	72.60±20.3	97.32±10.7
Mild to moderate	12 (7.9%)	7/5 (58/42%)	8.4±5.5	16.2±12.0	5.5±1.3	84.95±8.1	92.16±12.1
Moderate	32 (21.1%)	21/11 (66/34%)	7.6±4.4	11.8±9.4	8.3±4.1	79.70±17.3	92.89±14.2
Moderate to severe	25 (16.4%)	16/9 (64/36%)	8.3±6.2	12.8±10.4	10.8±7.5	70.84±19.7	84.67±12.2
Severe	39 (25.6%)	31/8 (79/21%)	7.4±5.0	12.8±11.1	11.8±7.6	71.90±16.4	88.69±17.5
Rigid	34 (22.3%)	27/7 (80/20%)	6.9±4.8	28.9±26.1	12.1±5.2	64.97±19.7	81.80±13.5

Clinical outcomes

In 101 patients (92.7%) and 142 feet (93.4%), we had a clinical follow-up at a mean age of 7.39±4.9 years for the patients (feet: 7.41±4). Accentuated intoeing during walking was noticed in 19 patients (18.6%) and 25 feet (16.4%). 6 patients (5.9%) and 10 feet (6.6%) demonstrated hindfoot varus, and 28 patients (27.5%) and 38 feet (25%) demonstrated residual forefoot adduction. The ROM of the ankle joint and the subtalar joint was good (Table 2).

**Table 2:** Clinical outcome Evaluation of the range of motion of ankle and subtalar joint.

	Ankle joint N (%)	Subtalar joint N (%)
Normal	75 (52.8%)	127 (89.4%)
Good	63 (44.4%)	10 (7.0%)
Poor	4 (2.8%)	5 (3.5%)

Function and health-related quality of life

At a mean follow-up of 10.0±6.2 years after surgery, 88 patients (82.2%) returned our questionnaires, which included the DSI and PedsQL. This corresponds to 126 feet (82.9%) with a follow-up of a mean 10.5 years.

The mean DSI was 74.8±17.5 (feet 79.5±17.5). The mean overall PedsQL™ was 87.8±12.6 (feet: 86.7±13.5). The PedsQL™ functional component showed a mean of 89.4±15.6 (feet: 90.9±17.1) and the PedsQL™ social component a mean of 86.9±13.5 (feet: 86.0±14.2). There is a trend to lower scores with more severe initial deformity (Table 2). 

The only risk factors were the age at treatment start, which seemed to be the most important risk factor, and the duration of day splinting.. When we compared our results with the literature, we found a medium-to-small effect size for the difference of the mean DSI score. Multiple linear regression model of potential risk factors is shown in Table 3.

**Table 3:** Multiple linear regression model of potential risk factors. The only risk factors were the age at treatment start, which seemed to be the most important risk factor, and the duration of day splinting. SE: standard error; LL: lower limit; UL: upper Limit; CI: confidence interval.

			95% CI			
Variables	Beta	SE	LL	UL	ß	p-value
Classification	-1.883	1.199	-4.267	0.502	-0.152	0.120
Duration of night splinting	-0.085	0.518	-1.115	0.945	-0.020	0.870
Duration of day splinting	-0.348	0.101	-0.549	-0.146	-0.333	0.001
Age at treatment start	5.028E-08	0.000	0.000	0.000	0.465	0.000
Number of preoperative casts	0.468	0.241	-0.011	0.947	1.092	0.056
Age at surgery	-10.844	7.478	-25.720	4.033	-0.831	0.151

Recurrence

Recurrence was defined as needing a second treatment, meaning a second round of casting or a second surgery. One of these treatments was required in 26.2% (28 patients) and 25.0% (38 feet), respectively. Five feet were treated with cast and orthosis only, four feet required a talar notchplasty due to anterior impingement of the ankle joint, four patients had a Achilles tendon lengthening and soft tissue release, 20 patients needed a bone correction of the midfoot with an osteotomy and in 5 patients a tibial derotation osteotomy was performed.

## Discussion

Our modification of the Ponseti method allows us to reduce the splinting time to two years with similar clinical and patient reported outcomes.

Night splinting period treatment

The Ponseti concept is the mainstay of treatment for idiopathic clubfeet [3]. The extended time of bracing of five to six years leads to impaired quality of life due to discomfort of the young patients [8,13]. 

Our study showed that a reduction of the mean time of bracing to 2.1 years was possible. Studies favoring the Ponseti concept argue that a reduction of night splinting years would result in less favorable outcomes [13]. However, our series clearly illustrates that the significant reduction in splinting years did not compromise long-term results in terms of patient-reported outcome (DSI), quality of life score (PedsQL™), or recurrence rate. This is even more important as several authors have demonstrated that compliance with bracing is an important issue. Alves showed that 34% to 61% of parents are non-compliant with bracing [13]. These children are reported to have a recurrence rate of 5 to 17 times higher than compliant patients. This finding was confirmed by Richards et al., who analyzed the time aspects of brace wearing in 124 children [14]. By the 18th month of brace wear, one in three patients wore the brace for less than 6 hours per day [7] compared to the recommended time of 10 hours per day [1]. Hence, we tried to adapt the original Ponseti method to shorten the time of bracing significantly. Applying our modified technique, the post-interventional time of bracing decreased to 2.1 years on average. This compares favorably to the four years of bracing that is recommended by many other authors performing percutaneous Achilles tenotomy [1,15]. In contrast, Ippolota et al. recommended a bracing time of three years after posteromedial release and 4 years for a posterior release [16].

Unilateral bracing

Moreover, our method allowed a unilateral brace in 69% of cases. The decision to choose the unilateral brace for the splinting period was made intraoperatively depending on the flexibility and the correction of the foot. The unilateral orthosis appears to be much more convenient compared to the Denis Browne splint, improving both patient comfort and compliance [8,17]. Our experience is similar to that of McCartney et al. with positive feedback after testing their new unilateral brace [8]. This was also confirmed by Berger et al., who found 91% compliance with a unilateral brace and only 46% compliance with the Denis Browne splint [17].

Patient-reported outcome and health-related quality of life

When comparing our method to the traditional Ponseti method, we found the patient-reported outcomes to be as good as previous reports. Our mean DSI of 74.8 is almost identical to the study by Symeonidis et al., where a mean DSI of 74 was reported after a 16-year follow-up [18,19]. Not only does this compare well to the published literature, but with a response rate of over 80% at 10-year follow-up, we feel these results are valid and significant.

**Figure 4:**
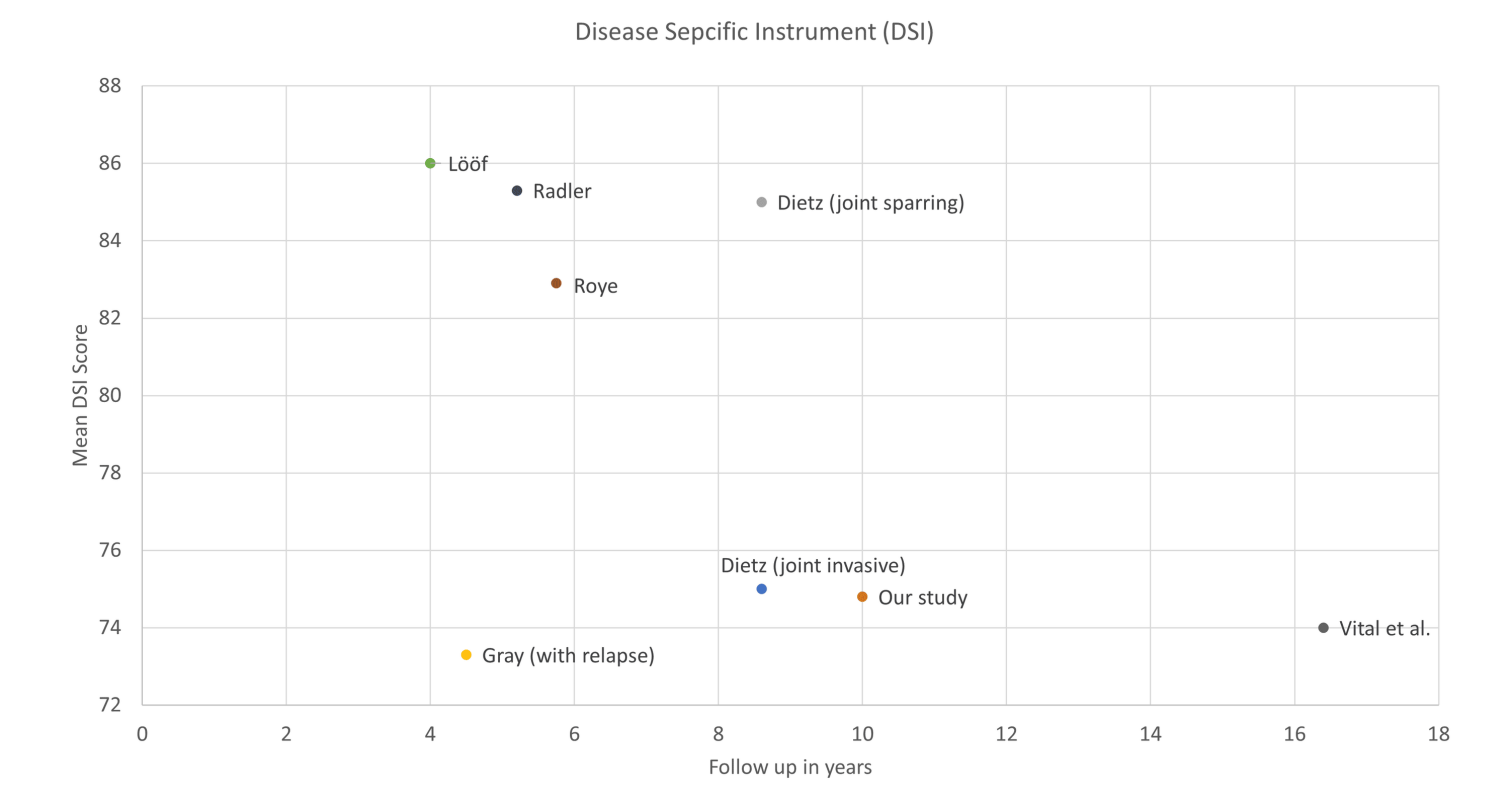
Disease specific instrument. Comparison of the mean DSI results of different studies depending on the time of follow up.

Our results are lower, however, when compared to studies with shorter follow-ups. For example, Gray et al. reported a mean DSI of 86.0 at 4 years follow-up, Roye et al. reported a mean DSI of 82.9 at 5.2 years follow-up, and Dietz et al reported a mean DSI of 80.0 at 8.6 years of follow-up [10,20,21] (Table 4 and Figure 4). Calculating the effect size with Cohen's d, the difference to our study is moderate. For the studies with longer follow-ups, the difference in the effect size is low. The shorter follow-up period may be a significant contributor to these scores since younger children may be less prone to complain about restraints when performing high-impact activities and sports. This might represent a limitation of the DSI, which is not adjusted for age or length of follow-up. In fact, Thomas et al. have found in their systematic review that functional outcomes like relapse rate positively correlate with length of follow-up [22].

**Table 4:** Review of literature. Comparison of the splinting time, outcome measurement, relapse and revision rate and time of follow up; NR: not reported.

Study	Treatment	Day spliniting time	Night splinting time only	Follow-up time	DSI score	PedsQL™	Relapse	Re-operation
Our study population	Limited posterior release	7.8 months	25.1 months	10.0 years	74.8	87.8	25%	22.4%
Dietz et al. [21]	Joint sparring	NR	NR	8.6 years	85.0	80.4	NR	NR
	Joint invasive	NR	NR	8.6 years	75.0	80.4	NR	NR
Gelfer et al. [23]	Review of literature	NR	NR	>4 years	NR	NR	32%	20.1%
Gray et al. [20]	Patients with relapse before tibialis anterior tendon transfer	NR	NR	53 months	73.3	NR	NR	NR
	Patients without relapse	NR	NR	48 months	86.0	NR	NR	NR
Haft et al. [24]	Ponseti technique	3 months	2 years	35 months	NR	NR	41%	27%
Ippolito et al. [16]	posteromedial release	NR	3 years	25 years	NR	NR	46.8%	46.8%
	posterior release	NR	4 years	19 years	NR	NR	40.8%	NR
Janicki et al. [15]	Ponseti technique	3 months	4 years	37 months	NR	NR	18.5%	8.2%
Lööf et al. [25]	Ponseti technique	2-3 months	4-5 years	9.5 years	83.0	NR	NR	NR
Radler et al. [2]	Ponseti technique	NR	NR	5.2 years	85.3	NR	30%	23%
Richards et al. [7]	Ponseti technique	3 months	2 years	4.3 years	NR	NR	37%	24.7%
Roye et al. [10]	Various	NR	NR	5.75 years	82.9	NR	35%	35%
Vitale et al. [18]	Surgical	NR	NR	16.4 years	74.0	NR	NR	NR

PedsQL™ was used to assess the influence of our treatment on quality of life. With a mean score of 87.8, we observed better results than those reported by Dietz et al. (mean 80.4) [20]. To our knowledge, no other study has reported on PedsQL™ for clubfoot treatment.

Clinical outcomes

At an average clinical follow-up of 7.4 years, 55% of our patients exhibited ankle dorsiflexion of more than 15 degrees, and another 40% showed ankle dorsiflexion of 0-15 degrees. In a systematic review, Rastogi et al. analyzed 12 studies that treated idiopathic clubfoot by the Ponseti method and had a follow-up of more than 10 years [26]. Eight of these studies reported an average ankle dorsiflexion of 10 degrees [26]. This is equivalent to our series suggesting that open release of the ankle joint at three months is not associated with decreased ROM. ROM is an important aspect of patient satisfaction, as better dorsiflexion is correlated with better function [10]. Analysis of subtalar joint motion is more difficult as there is less reported data. Smith et al. showed decreased mobility of the subtalar joint for both surgical treatment and treatment according to Ponseti [27]. This was not observed during the clinical examination of our study population.

Correct hindfoot alignment is an important aspect to achieve good foot function [10]. With only 7% of residual varus in our study, this is only slightly better than the results reported by Roye et al., who found 9% of heel varus in 46 patients at 45 months after surgical correction [10]. Residual varus is well known to occur after Ponseti treatment and has a dynamic component during gait [27].

Our study population showed a slightly higher forefoot adduction, with 26.7% in comparison to the 18% in the study of Radler et al. [2]. This might be due to the use of a unilateral orthosis, which is considered to provoke a risk of residual forefoot adduction and external rotation [16]. Residual abduction is a common problem after Ponseti treatment and can be treated by tibialis anterior transfer. It was the most common revision surgery performed in 13% of cases by Radler et al. [2]. In contrast to this, we only performed the transfer in 3.3% of revision cases. 

Limitations

Our study might have some limitations. First, it is a retrospective case series. Therefore, it is subject to methodological issues that all retrospective studies share. Since a control group is lacking a comparison to other treatment protocols or the natural history of the cases, it is difficult and is possible only comparing our results with the results of published studies. Our study results align well with existing literature, suggesting a strong association between our modification and a reduced splinting period. However, due to methodological limitations, this association cannot be considered causative. Additionally, the final choice of operative technique was determined intraoperatively by the surgeon (flowchart, Figure 2). While all treating physicians were trained at the same institution and had access to the flowchart, the treatment plan was not a strictly standardized operating procedure. Hence, some cases might have been treated differently if another surgeon would have performed the identical surgery in the same patient. All our limitations are frequent in studies assessing club foot treatment.

## Conclusions

The modification of the Ponseti method by performing both an ATL and a limited posterior release leads to a shortening of the splinting period by more than 50% from an average of five years to two years. In addition, a unilateral brace appears to be sufficient in two-thirds of patients. Furthermore, this reduction in splinting time occurred without limiting clinical results and patient-reported outcomes. Prospective-controlled trials or cohort studies would further help strengthen these findings.
